# Virtual Mice, Real Errors: A Sensor-Aware Generative Framework for In Silico Ethology

**DOI:** 10.3390/s26102977

**Published:** 2026-05-09

**Authors:** Reza Sayfoori, Goli Vaisi, Hung Cao

**Affiliations:** 1 Department of Electrical Engineering and Computer Science, University of California, Irvine, Irvine, CA 92697, USA; gvaisi@uci.edu (G.V.); hungcao@uci.edu (H.C.); 2 Department of Biomedical Engineering, University of California, Irvine, Irvine, CA 92697, USA; 3 Department of Computer Science, University of California, Irvine, Irvine, CA 92697, USA

**Keywords:** computational ethology, digital twin, generative modeling, semi-Markov model, trajectory generation, ultra-wideband (UWB), line-of-sight (LoS), non-line-of-sight (NLoS), domain shift, sensor-aware modeling

## Abstract

Long-duration animal trajectories are central to computational ethology, yet constructing large rodent cohorts remains costly, time-intensive, and constrained by animal-use considerations. We present a sensor-aware generative framework that separates latent behavioral dynamics from sensing-induced observation distortion to synthesize observed-domain trajectories that are behaviorally plausible while reproducing proxy-referenced observation distortions. The framework combines a run-level semi-Markov ethology model, occupancy calibration, and state-conditioned kinematic generation with a regime-dependent Ultra-Wideband observation channel that explicitly captures Line-of-Sight and Non-Line-of-Sight sensing conditions. Using four UWB sessions, this proof-of-concept study models three states—exploring, feeding, and burrowing—and evaluates realism through state occupancy, state-conditioned kinematic divergence, residual-domain agreement, and mean-squared displacement across time lags. We further assess whether sensor-aware conditioning improves robustness under LoS/NLoS domain shift in downstream trajectory classification. Sensor-aware conditioning yields stable mixed-domain performance with AUC = 0.995, whereas condition-agnostic baselines decline to AUC = 0.974 and AUC = 0.901. These results support the feasibility of sensor-aware in silico ethology as a proof-of-concept framework for controlled robustness studies and algorithm evaluation under proxy-referenced observation distortion. Because the present evaluation is based on four UWB sessions and uses a smoothed UWB-derived reference trajectory rather than independent ground truth, broader applications to synthetic-cohort generation, disease modeling, and statistical power-analysis workflows should be considered future directions requiring validation in larger datasets.

## 1. Introduction

Quantitative rodent phenotyping plays a central role in neuroscience and neuroengineering because many disease models manifest as subtle, time-varying alterations in locomotion, exploration, and state-dependent behavioral dynamics that are not well captured by short assays or coarse summary endpoints [1,2]. Reliable inference at scale, however, is limited by two persistent bottlenecks. First, assembling sufficiently large and well-controlled cohorts is expensive, labor-intensive, and constrained by animal-use considerations. Second, behavioral measurement is inherently imperfect: sensing modality, hardware configuration, and deployment conditions can systematically bias derived kinematic features and, consequently, downstream inference. When the sensing process is not explicitly modeled, such distortions can confound behavioral interpretation and obscure the underlying ethological signal [3,4].

Markerless video pipelines have substantially advanced high-throughput behavioral quantification by enabling the extraction of trajectories and pose features from naturalistic experiments [5]. Despite these advances, video-based systems remain sensitive to occlusion, illumination variability, cage geometry, and camera placement, and long-duration monitoring often requires stable visibility and tightly controlled imaging conditions [5,6]. Beyond experimental tracking, recent work in generative trajectory modeling, synthetic behavioral data generation, digital twins, and sim-to-real learning has shown that simulation can provide a useful testbed for evaluating algorithms under controlled variability [7,8,9]. However, many existing simulation approaches emphasize latent motion plausibility while treating the sensing process as either idealized or weakly specified. In contrast, the present work focuses on explicitly coupling behavioral trajectory generation with a sensing-regime-dependent observation channel, allowing LoS/NLoS-specific proxy-referenced observation distortion to be represented as part of the generative process.

Ultra-Wideband (UWB) real-time localization offers a complementary alternative by enabling continuous planar tracking without reliance on direct visual access and by providing greater robustness to multipath effects than conventional narrowband radio-frequency systems [10,11]. However, UWB observations are strongly regime-dependent. Under Non-Line-of-Sight (NLoS) propagation, range estimates may exhibit systematic bias, heavy-tailed errors, and intermittent outlier events, all of which distort reconstructed trajectories and alter the distributions of derived motion features [11,12]. This induces a domain shift between Line-of-Sight (LoS) and NLoS recordings, thereby degrading cross-condition generalization and complicating ethological interpretation when sensing is treated merely as a nuisance perturbation rather than as part of the generative process itself [12,13]. These considerations motivate a sensor-aware trajectory generator that explicitly separates latent ethology from a regime-dependent observation channel, thereby enabling synthetic trajectories that are ethologically plausible while reproducing proxy-referenced LoS/NLoS observation distortions within the scope of the available UWB data.

### 1.1. From Descriptive Ethology to In Silico Ethology

Rare, heterogeneous, or slowly progressing disease models often require long-duration monitoring and comparatively large cohorts to achieve adequate statistical power, making behavioral data collection costly, time-intensive, and experimentally restrictive. In addition to this logistical burden, repeated handling and procedural intervention may introduce physiological and behavioral confounds that affect the validity of downstream inference. For example, anesthesia and sedation can alter behavioral state and physiological stability, thereby complicating the interpretation of experimental behavioral measurements [14]. Such considerations are directly aligned with the 3Rs framework (Replacement, Reduction, and Refinement), particularly the principles of Reductionand Refinement, which prioritize methodological strategies that decrease the number of live animals required while minimizing experimental burden and improving the quality of collected data [3].

Motivated by these constraints, we advance a sensor-aware generative framework for in silico ethology: a probabilistic simulator designed to generate behaviorally plausible observed-domain synthetic trajectories while explicitly modeling how sensing regime (LoS versus NLoS) transforms latent motion into sensor-observed tracks. The proposed formulation is aligned with the broader digital twin paradigm, in which a virtual representation captures both intrinsic system dynamics and the measurement processes through which those dynamics are observed [8,15]. In the present study, this perspective is instantiated at the trajectory level through an explicit observation channel, allowing the generator to produce synthetic data in the same observed data domain used by downstream inference. We evaluate the framework through statistical realism checks and downstream robustness assessment under LoS/NLoS domain shift.

**Relationship to Prior Work.** This study builds directly on our two prior lines of work on UWB-based rodent tracking and probabilistic behavioral analysis [10,16]. In the first, we established the sensing platform itself by validating a high-precision UWB real-time locating system against synchronized video and quantifying localization performance under both LoS and NLoS conditions. In the second, we showed that sensing quality propagates into higher-level behavioral summaries by linking LoS/NLoS signal integrity to entropy-, Bernoulli-, and Markov-based analyses of behavioral dynamics.

The present work addresses a distinct objective. Rather than characterizing sensing performance alone or interpreting observed trajectories through probabilistic summaries, we develop a sensor-aware generative framework for in silico ethology. The key step is to factorize trajectory generation into a latent behavioral process and a regime-dependent observation channel. This enables the synthesis of observed-domain synthetic trajectories that preserve ethological structure while reproducing LoS/NLoS-specific proxy-referenced observation distortion, thereby providing a controlled basis for preliminary synthetic-cohort generation, robustness studies, and stress-testing of downstream inference under observation-domain shift, rather than a fully validated virtual-cohort or disease-modeling platform. The principal contribution of this paper is therefore not another descriptive analysis of UWB trajectories but a factorized generative model that links behavioral simulation to sensing-aware observation modeling.

**Figure 1:**
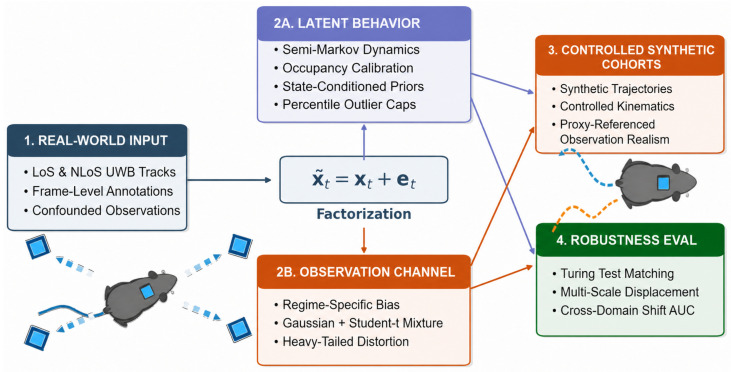
Conceptual overview of the proposed sensor-aware generative framework for in silico ethology. Real UWB rodent trajectories acquired under Line-of-Sight (LoS) and Non-Line-of-Sight (NLoS) conditions are factorized into a latent behavioral component and a regime-dependent observation channel. The latent branch models ethological dynamics through semi-Markov state evolution, occupancy calibration, and state-conditioned kinematic priors, whereas the observation branch captures proxy-referenced sensing bias and heavy-tailed observation distortion. These components are recombined to generate observed-domain synthetic trajectories and controlled synthetic cohorts, which are subsequently evaluated using statistical realism checks, temporal realism, and robustness under LoS/NLoS domain shift.

Figure 1 provides a conceptual roadmap of the proposed framework before the formal development in Section 2. Starting from real UWB trajectories acquired under LoS and NLoS conditions, the framework factorizes the problem into a latent behavioral branch and a regime-dependent observation branch. The latent branch models ethological structure through semi-Markov state dynamics, occupancy calibration, and state-conditioned kinematic priors, whereas the observation branch captures proxy-referenced sensing distortion through a condition-dependent UWB channel. These two components are then recombined to generate observed-domain synthetic trajectories that are behaviorally plausible at the latent level while reproducing proxy-referenced observation distortions in the observed UWB domain. Because the present study is based on a limited four-session UWB dataset, the framework is evaluated as a proof-of-concept demonstration rather than as a fully validated general-purpose behavioral simulator. The remainder of the manuscript formalizes this pipeline and evaluates it using statistical realism checks, temporal realism, and downstream robustness under LoS/NLoS domain shift.

### 1.2. Contributions

We introduce a sensor-aware generative framework for in silico ethology that separates latent behavioral dynamics from sensing-induced distortion. The main contributions are as follows:

- Sensor-aware factorization: Trajectory generation is formulated as a two-stage process that couples latent behavioral dynamics with a regime-conditioned observation channel.
- Semi-Markov behavioral modeling with occupancy calibration: Behavioral state evolution is modeled using a semi-Markov process whose transition structure is calibrated to match empirical frame-level occupancy.
- State-conditioned kinematic priors with tail control: Step length and turning angle are sampled from state-conditioned priors with quantile-based caps to improve stability in long-horizon synthesis.
- Temporal realism beyond marginal statistics: Temporal structure is evaluated through mean-squared displacement (MSD) across time lags and summarized by the RMSE between real and synthetic curves.
- Statistical realism across sensing regimes: Realism is quantified through occupancy agreement, state-conditioned kinematic divergence, and residual-domain agreement under LoS and NLoS conditions.
- Downstream robustness testing under domain shift: Conditioning on sensing regime improves mixed-domain classification in a controlled synthetic benchmark, whereas condition-agnostic baselines degrade under LoS/NLoS shift.

Guided by the conceptual workflow in Figure 1, Section 2 formalizes the dataset representation, latent semi-Markov behavioral model, state-conditioned kinematic generator, and regime-dependent observation channel used throughout this study.

## 2. Methods and Implementation

### 2.1. Sensor-Aware Generative Formulation

Because the UWB hardware platform, synchronization pipeline, and baseline LoS/NLoS sensing characteristics were established in our prior studies [10,16], the present section focuses only on the components required to specify the proposed sensor-aware generative framework and its evaluation. We model in silico ethology by explicitly separating latent behavioral dynamics from sensing-regime-dependent observation distortion. At the trajectory level, the latent behavioral state $z_{t}$ governs the evolution of an ideal planar position $x_{t}$, which is then mapped to an observed UWB position ${\tilde{x}}_{t}$ through a condition-dependent observation channel. The resulting causal structure is

(1) $$z_{t}\;\to \;x_{t}\;\to \;{\tilde{x}}_{t}$$

Here, $z_{t}\in Z$ denotes the discrete behavioral state, and $x_{t},{\tilde{x}}_{t}\in R^{2}$ denote latent and observed planar positions, respectively, expressed in Meters.

To model the sensing process, we assume that the observed position is generated by an additive distortion applied to the latent trajectory:

(2) $${\tilde{x}}_{t}=x_{t}+e_{t}$$

where $e_{t}\in R^{2}$ denotes the observation distortion. This distortion is conditioned on the sensing regime c∈{LoS,NLoS} and is expected to exhibit regime-specific behavior, including systematic bias and heavier tails under NLoS propagation [11,12,13]. To capture these effects, we model the distortion using a state- and regime-conditioned location-plus-mixture representation:

(3) $$e_{t}|\left(z_{t}=i,c\right)=\mu_{i,c}+u_{t}$$

where $\mu_{i,c}\in R^{2}$ is a state- and regime-dependent bias term and $u_{t}\in R^{2}$ is a zero-centered stochastic distortion component. We model $u_{t}$ as

(4) $$u_{t}∼\left(1-\rho_{i,c}\right)\;N\left(0,\Sigma_{i,c}\right)+\rho_{i,c}\;T_{\nu }\left(0,\Lambda_{i,c}\right)$$

where $\rho_{i,c}\in \left[0,1\right]$ denotes the outlier probability, $\Sigma_{i,c}\in R^{2\times 2}$ is the nominal covariance matrix associated with the Gaussian component, and $T_{\nu }\left(0,\Lambda_{i,c}\right)$ denotes a multivariate Student-*t* distribution with degrees of freedom ν and scale matrix $\Lambda_{i,c}\in R^{2\times 2}$ [17]. This mixture structure is designed to capture both nominal localization variability and the heavy-tailed deviations that arise under adverse sensing conditions.

Model parameters are estimated from residuals defined by

(5) $$e_{t}={\tilde{x}}_{t}-x_{t}$$

following the estimation procedure described in Section 2.5.4. This factorization preserves $x_{t}$ as the ethology-driven latent trajectory while allowing ${\tilde{x}}_{t}$ to reproduce sensing-regime-specific distortions observed in real UWB recordings. As a result, the proposed framework provides a principled mechanism for generating trajectories that are behaviorally plausible while reproducing sensing-regime-specific, proxy-referenced observation distortions.

### 2.2. Dataset, State Space, and Notation

The present study uses a previously curated UWB trajectory dataset derived from four experimental sessions recorded under two sensing regimes, with two sessions acquired under LoS and two under NLoS conditions [10,16]. Each session provides a planar observed trajectory $\left\{{\tilde{x}}_{t}\right\}$ together with a frame-level behavioral label sequence $\left\{z_{t}\right\}$. These labels are not newly inferred here; they are treated as fixed annotations and are used only for state-dynamics estimation, occupancy calibration, and realism evaluation. All sessions are sampled at Δt=0.1s and contain $T_{\mathrm{real}}$ synchronized frames after preprocessing.

Let ${\tilde{x}}_{t}={\left[{\tilde{x}}_{t},{\tilde{y}}_{t}\right]}^{⊤}\in R^{2}$ denote the observed planar UWB position in Meters at discrete time index $t\in \{1,\ldots ,T_{\mathrm{real}}\}$. We further define $x_{t}={\left[x_{t},y_{t}\right]}^{⊤}\in R^{2}$ as the corresponding reference trajectory, representing the least-distorted estimate of the animal’s motion and used to parameterize ethological priors and residual statistics (Section 2.5.1). All positions are expressed in a common planar coordinate system in Meters; a rigid translation is applied such that the arena origin coincides with (0,0). The discrete behavioral state space is defined as  

(6) Z={1,2,3}≡{exploring,feeding,burrowing}.

This reduced three-state formulation differs from our earlier four-state descriptive analysis in [16], which included resting in addition to exploring, feeding, and burrowing. In the present study, the objective is not to reproduce the full prior taxonomy one-to-one, but to obtain a stable generative model under a limited-data regime. We therefore restrict the latent state space to behaviors with stronger trajectory-defining kinematic structure and more reliable run-level estimation from the available sessions. Relative to the active states considered here, resting is dominated by near-zero motion and contributes limited additional spatial dynamics, while its inclusion increases sparsity in dwell-time and transition estimates for the semi-Markov generator. The resulting three-state space is thus adopted to improve robustness of occupancy calibration, duration modeling, and state-conditioned trajectory synthesis, while preserving the principal ethological modes needed for the sensor-aware generative objective of this work. Nevertheless, resting and immobility are biologically important in many rodent phenotyping and neurological disease contexts; therefore, excluding resting limits the behavioral scope and generalizability of the present proof-of-concept framework.

Empirical “real” statistics are computed from temporally aligned position–label streams obtained via nearest-neighbor synchronization. These statistics serve as the reference distribution for all subsequent comparisons between real and synthetic trajectories. The run-level representation used to model behavioral dynamics, based on a semi-Markov formulation, is introduced in Section 2.3.

### 2.3. Run Segmentation and Semi-Markov Dynamics

Using the notation introduced in Section 2.2, we transform the frame-level behavioral label sequence ${\left\{z_{t}\right\}}_{t=1}^{T_{\mathrm{real}}}$ into a run-level representation by collapsing consecutive identical labels into segments. This representation removes explicit self-transitions and is therefore well suited to semi-Markov modeling, in which state persistence is captured through an explicit duration process rather than repeated self-transition probabilities [18,19].

#### 2.3.1. Run Representation

Let the run boundaries be defined by

(7) $$1=\tau_{1}<\tau_{2}<\ldots <\tau_{K}\leq T_{\mathrm{real}}$$

such that the state label $z_{t}$ remains constant on each interval $\left[\tau_{k},\tau_{k+1}-1\right]$. The *k*-th run is then represented by the pair

(8) $$\left(s_{k},d_{k}\right),\;s_{k}=z_{\tau_{k}}\in Z,\;d_{k}=\tau_{k+1}-\tau_{k}\in N$$

where $\tau_{K+1}=T_{\mathrm{real}}+1$ by convention. Here, $s_{k}$ denotes the behavioral state associated with the *k*-th run, and $d_{k}$ denotes its duration measured in Frames. By construction, the run durations satisfy

(9) $$\sum_{k=1}^{K}d_{k}=T_{\mathrm{real}}$$

This run-level representation provides the foundation for the semi-Markov state model developed in the following subsections [18,19].

#### 2.3.2. Run-Level Markov Chain Estimation

Given the run sequence in Equation (8), we model the succession of run states $\left\{s_{k}\right\}$ as a first-order Markov chain with transition matrix $P\in {\left[0,1\right]}^{\left|Z|\times |Z\right|}$:

(10) $$P_{ij}≜P\left(s_{k+1}=j|s_{k}=i\right),\;\sum_{j\in Z}P_{ij}=1.$$

Let $N_{ij}$ denote the observed number of run-level transitions from state *i* to state *j*, aggregated across all real sessions [20]. The corresponding maximum-likelihood estimate is

(11) $${\hat{P}}_{ij}=\frac{N_{ij}}{\sum_{j^{\prime }\in Z}N_{ij^{\prime }}}$$

Because consecutive identical labels are collapsed into runs, self-transitions are not represented explicitly at the run level. We therefore impose

(12) $${\hat{P}}_{ii}=0,\;i\in Z,$$

and renormalize each row over j≠i so that persistence is modeled exclusively through the dwell-time distribution.

#### 2.3.3. State-Conditioned Dwell-Time Model

State persistence is modeled through an explicit run-level dwell-time distribution. For each behavioral state i∈Z,

(13) $$d|\left(s=i\right)∼D_{i}$$

where $D_{i}$ is the empirical distribution of observed run durations $\left\{d_{k}:\;s_{k}=i\right\}$. The corresponding mean dwell time is

(14) $$m_{i}≜E\left[d|s=i\right]$$

estimated from run durations measured in Frames. During synthesis, $D_{i}$ determines how long the process remains in state *i* before transitioning according to the run-level Markov chain.

#### 2.3.4. Frame-Level Occupancy Induced by Run Dynamics

Let $\mu \in R^{\left|Z\right|}$ denote the stationary distribution of the run-level Markov chain:

(15) $$\mu^{⊤}=\mu^{⊤}P,\;1^{⊤}\mu =1.$$

Because synthesis is defined over runs rather than frames, frame-level occupancy depends on both run visitation frequency and mean dwell time. If $m_{i}$ is the mean dwell time of state *i* from Equation (14), then the induced frame-level occupancy is

(16) $$\pi_{i}=\frac{\mu_{i}m_{i}}{\sum_{\ell \in Z}\mu_{\ell }m_{\ell }}$$

which links run-level dynamics to the expected fraction of frames spent in each state [19]. This relation is used directly in the occupancy calibration step of Section 2.4.

### 2.4. Occupancy Calibration to Match Real Ethological Statistics

Let $\pi^{\mathrm{real}}\in R^{\left|Z\right|}$ denote the empirical frame-level occupancy measured from the real data. For each state i∈Z, this quantity is defined as  

(17) $$\pi_{i}^{\mathrm{real}}≜\frac{1}{T_{\mathrm{real}}}\sum_{t=1}^{T_{\mathrm{real}}}I\left\{z_{t}=i\right\}$$

where I{·} denotes the indicator function. Thus, $\pi_{i}^{\mathrm{real}}$ represents the empirical fraction of frames assigned to state *i* in the observed dataset.

Direct estimation of the run-level transition matrix from empirical run counts can lead to unstable occupancy at the frame level, particularly when one behavioral state dominates the sequence through long dwell times. In the present dataset, exploring is the dominant state and is characterized by long runs. As a result, the number of observed run-level exits from this state is small, which in turn makes the corresponding outgoing transition probabilities sensitive to sampling variability. To improve stability while preserving the empirical structure of the rarer states, we calibrate only the outgoing distribution of the dominant run state (i=1, corresponding to exploring; see Equation (6)) and retain the empirical transition structure for the remaining states. Because persistence is modeled separately through dwell-time distributions in Section 2.3.3, the run-level chain is defined without self-transitions, i.e., $P_{ii}=0$, and each row is normalized over j≠i.

Accordingly, we define a calibrated transition matrix P(α) of the form

(18) $$P\left(\alpha \right)=\left[\begin{array}{ccc} 0 & \alpha_{2} & \alpha_{3}\\ \beta_{21} & 0 & \beta_{23}\\ \beta_{31} & \beta_{32} & 0 \end{array}\right],\;\alpha_{2}+\alpha_{3}=1,\;\alpha_{2},\alpha_{3}\geq 0,$$

where $\alpha =[\alpha_{2},\alpha_{3}]$ parameterizes the calibrated outgoing transition distribution from exploring. The remaining off-diagonal entries are obtained by renormalizing the empirical transition estimates:

(19) $$\beta_{ij}≜\frac{{\hat{P}}_{ij}}{\sum_{j^{\prime }\in Z\setminus \left\{i\right\}}{\hat{P}}_{ij^{\prime }}},\;i\in \left\{2,3\right\},\;j\in Z\setminus \left\{i\right\}.$$

This construction preserves the empirically observed transition structure of the rare states while allowing controlled adjustment of the dominant state’s outgoing behavior.

For any admissible choice of α, we compute the stationary run distribution μ(α) using Equation (15) and then obtain the induced frame-level occupancy π(α) via Equation (16). The calibration parameters are selected by minimizing the discrepancy between the model-implied occupancy and the empirical occupancy observed in the real data:

(20) $$\begin{array}{cc} \hat{\alpha } & =\arg\min_{\alpha_{2},\alpha_{3}}∥\pi \left(\alpha \right)-\pi^{\mathrm{real}}{∥}_{2}^{2},\\ \mathrm{subject}\;\mathrm{to}\;\; & \alpha_{2}+\alpha_{3}=1,\;\alpha_{2}\geq 0,\;\alpha_{3}\geq 0. \end{array}$$

Because the optimization is two-dimensional, it can be solved efficiently by grid search. The resulting calibration preserves the transition structure of the rare states while matching the empirical frame-level occupancy of the observed ethological data more closely.

### 2.5. State-Conditioned Kinematic Model

#### 2.5.1. Reference Trajectory Construction

Let ${\tilde{x}}_{t}={\left[{\tilde{x}}_{t},{\tilde{y}}_{t}\right]}^{⊤}\in R^{2}$ denote the observed UWB position under sensing regime c∈{LoS,NLoS}. Because an external synchronized ground-truth trajectory, such as video-based tracking, is not used for model fitting, we define the reference trajectory $x_{t}={\left[x_{t},y_{t}\right]}^{⊤}$ as a smoothed UWB-derived proxy rather than as an independent ground-truth trajectory. Consequently, the residuals used for observation-channel estimation should be interpreted as deviations from this denoised UWB proxy, not as true localization errors relative to an external reference. This proxy is used only to parameterize ethological kinematic priors and to estimate residual statistics for the observation channel. Specifically, $x_{t}$ is obtained by applying a two-stage smoothing operator to each coordinate:

(21) $$R_{w}\left(s_{t}\right)≜{\mathrm{MA}}_{w}\;\left({\mathrm{Med}}_{w}\left(s_{t}\right)\right)$$

where ${\mathrm{Med}}_{w}\left(\;\cdot \;\right)$ denotes a centered rolling median, ${\mathrm{MA}}_{w}\left(\;\cdot \;\right)$ denotes a centered rolling mean, and *w* is the smoothing-window length measured in Samples. In the present study, we use w=21 under LoS and w=41 under NLoS to account for the larger magnitude and frequency of outliers observed under NLoS conditions. These window lengths were selected once and held fixed throughout all analyses to ensure consistency across experiments.

The reference trajectory is then defined coordinate-wise as

(22) $$x_{t}=R_{w}\left({\tilde{x}}_{t}\right),\;y_{t}=R_{w}\left({\tilde{y}}_{t}\right),$$

thereby yielding the reference position $x_{t}$ in Meters. For subsequent estimation, we define the residual distortion as

(23) $$e_{t}≜{\tilde{x}}_{t}-x_{t}$$

where $e_{t}\in R^{2}$ represents the discrepancy between the observed UWB position and the reference trajectory. These residuals form the basis for estimating the sensing-regime-dependent observation model described in Section 2.5.4.

#### 2.5.2. Feature Definitions

From each planar trajectory, we extract frame-to-frame displacement, step length, heading, and turning angle. For t≥2,

(24) $$\Delta x_{t}≜x_{t}-x_{t-1},$$

(25) $$\ell_{t}≜{∥\Delta x_{t}∥}_{2},$$

(26) $$\phi_{t}≜\mathrm{atan}2\left(\Delta y_{t},\Delta x_{t}\right),$$

and

(27) $$\theta_{t}≜\mathrm{wrap}\left(\phi_{t}-\phi_{t-1}\right)\in \left(-\pi ,\pi \right].$$

The same definitions are applied to the observed trajectory by replacing $x_{t}$ with ${\tilde{x}}_{t}$. In the proposed framework, state-conditioned motion priors are estimated from the reference features $\left(\ell_{t},\theta_{t}\right)$, whereas sensing effects are modeled separately through the residual process in Equation (23).

#### 2.5.3. State-Conditioned Priors

To model ethological dynamics independently of sensing artifacts, we construct state-conditioned kinematic priors from the reference trajectory $\left\{x_{t}\right\}$ using the features defined in Equations (25)–(27). For each behavioral state i∈Z, we collect the empirical samples

(28) $$L_{i}≜\left\{\ell_{t}:\;z_{t}=i\right\},\;T_{i}≜\left\{\theta_{t}:\;z_{t}=i\right\},$$

where $L_{i}$ denotes the set of step-length samples associated with state *i*, and $T_{i}$ denotes the corresponding set of turning-angle samples.

These state-conditioned sample sets define empirical priors over local motion increments. Because $\ell_{t}$ and $\theta_{t}$ are defined from the transition from frame t−1 to frame *t* (Equations (25)–(27)), conditioning is performed using the arrival-state label $z_{t}$. During synthesis, increments used to construct $\left(\phi_{t},x_{t}\right)$ are therefore sampled conditional on $z_{t}$ for t=2,…,T. Specifically,

(29) $$\ell_{t}|\left(z_{t}=i\right)∼\hat{p}\left(L_{i}\right),\;\theta_{t}|\left(z_{t}=i\right)∼\hat{p}\left(T_{i}\right),$$

where $\hat{p}\left(\;\cdot \;\right)$ denotes the empirical distribution induced by the corresponding sample set. This construction allows the latent generator to preserve state-specific motion characteristics directly from the observed behavioral data while remaining agnostic to sensing-regime-dependent distortion. To improve stability during long-horizon synthesis, sampled increments are further subject to percentile-based capping, as described in Section 2.5.5. The latent heading and position are then propagated using Equations (38) and (39), after which sensor-observed trajectories are generated via the observation model in Equation (2).

#### 2.5.4. Sensor-Aware Conditioning (Observation Channel)

Sensing effects are modeled as an explicit observation channel applied to the latent trajectory rather than being absorbed into the ethological priors. Let $c_{t}\in \left\{\mathrm{LoS},\mathrm{NLoS}\right\}$ denote the sensing regime at frame *t*. Using the residual process

(30) $$e_{t}={\tilde{x}}_{t}-x_{t}$$

we fit the state- and regime-conditioned mixture model in Equations (3) and (4). For each state–regime pair, the parameter set

(31) $$\Psi_{i,c}≜\left\{\mu_{i,c},\Sigma_{i,c},\rho_{i,c},\Lambda_{i,c}\right\}$$

is estimated by expectation–maximization. To stabilize estimation in sparse bins, the Student-*t* degrees of freedom ν are fixed (here ν=4), and covariance and scale updates are regularized with a small diagonal term.

When a given (i,c) bin contains fewer than $N_{\min}$ residual samples, we apply hierarchical pooling. Residuals are first pooled across behavioral states within the same sensing regime to estimate a condition-specific parameter set $\Psi_{c}$; if the pooled sample size remains below $N_{\min}$, a single global parameter set is estimated across all states and both regimes. In all experiments, we use $N_{\min}=5$.

During synthesis, observation distortion is sampled from the distribution associated with the active state–regime pair $\left(z_{t},c\right)$, optionally capped as described in Section 2.5.5, and added to the latent trajectory through Equation (2). This preserves latent behavioral structure while reproducing sensing-regime-specific distortion in the observed domain.

#### 2.5.5. Percentile Caps for Outlier Control

To improve stability during long-horizon synthesis in the presence of heavy-tailed kinematic increments and observation outliers, we apply quantile-based caps (Winsorization) to both (i) latent motion increments and (ii) sampled observation distortions.

**Latent increment caps:** For each behavioral state i∈Z, let
  (32) $$L_{i}=\left\{\ell_{t}:\;z_{t}=i\right\},\;A_{i}=\left\{\right|\theta_{t}\left|:\;z_{t}=i\right\},$$
  denote the empirical sample sets of step lengths and turning-angle magnitudes, respectively. We then define high-percentile caps
  (33) $$q_{\ell ,i}≜Q_{p_{\ell }}\left(L_{i}\right),\;q_{\theta ,i}≜Q_{p_{\theta }}\left(A_{i}\right),$$
  where $Q_{p}\left(\;\cdot \;\right)$ denotes the *p*-quantile operator, $q_{\ell ,i}$ is the step-length cap in Meters, and $q_{\theta ,i}$ is the turning-angle magnitude cap in Radians. Given sampled increments $\left(\ell_{t},\theta_{t}\right)$, the capped quantities are defined as
  (34) $$\ell_{t}^{\mathrm{cap}}=\min\left(\ell_{t},q_{\ell ,z_{t}}\right),\;\theta_{t}^{\mathrm{cap}}=\mathrm{sgn}\left(\theta_{t}\right)\min\;\left(|\theta_{t}|,q_{\theta ,z_{t}}\right),$$
  where $\ell_{t}^{\mathrm{cap}}$ and $\theta_{t}^{\mathrm{cap}}$ denote the capped step length and turning angle, respectively. In all experiments, we use $p_{\ell }=p_{\theta }=0.995$.

**Distortion caps:** To control extreme observation distortions, we apply an analogous procedure to residuals. For each state–regime pair (i,c), we define the residual magnitude
  (35) $$r_{t}≜{∥e_{t}∥}_{2}$$
  where $e_{t}={\tilde{x}}_{t}-x_{t}$ as defined in Equation (23). We then compute the high-percentile distortion cap
  (36) $$q_{e,i,c}≜Q_{p_{e}}\left(\{r_{t}:\;z_{t}=i,\;c_{t}=c\}\right)$$
  where $c_{t}\in \left\{\mathrm{LoS},\mathrm{NLoS}\right\}$ denotes the sensing regime associated with frame *t*, the quantile is evaluated separately within each state–regime subset, and $q_{e,i,c}$ is expressed in Meters. After sampling $e_{t}$ from the observation model in Equations (3) and (4), we cap its magnitude according to
  (37) $$e_{t}^{\mathrm{cap}}=e_{t}\;\cdot \;\min\;\left(1,\frac{q_{e,z_{t},c}}{∥e_{t}{∥}_{2}}\right)$$
  where $e_{t}^{\mathrm{cap}}$ denotes the capped distortion vector in Meters. In our experiments, we set $p_{e}=0.999$.

Given the capped latent increments $\left(\ell_{t}^{\mathrm{cap}},\theta_{t}^{\mathrm{cap}}\right)$, we propagate the latent heading and position according to

(38) $$\begin{array}{cc} \phi_{t} & \leftarrow \phi_{t-1}+\theta_{t}^{\mathrm{cap}},\;t=2,\ldots ,T, \end{array}$$

(39) $$\begin{array}{cc} x_{t} & \leftarrow x_{t-1}+\ell_{t}^{\mathrm{cap}}\left[\begin{array}{c} \cos(\phi_{t})\\ \sin(\phi_{t}) \end{array}\right],\;t=2,\ldots ,T, \end{array}$$

where $\phi_{t}$ is expressed in Radians and $x_{t}$ in Meters. Observed-domain synthetic positions are then generated by applying the additive observation model in Equation (2) using the capped proxy-referenced distortion term $e_{t}^{\mathrm{cap}}$.

### 2.6. Feeding Smoothing Constraint for Temporal Coherence

Because feeding is sparse and typically occurs in short runs, naive synthesis can occasionally produce isolated single-frame feeding events that are not representative of the real data. We therefore apply a deterministic smoothing operator to the generated state sequence:

(40) $${\tilde{z}}_{1:T}=S\left(z_{1:T}\right)$$

where S(·) removes singleton feeding runs. Specifically, for indices satisfying

(41) $$z_{t}=\mathrm{feeding},\;z_{t-1}\neq \mathrm{feeding},\;z_{t+1}\neq \mathrm{feeding},$$

the isolated label is reassigned to the adjacent non-feeding state associated with the longer neighboring run, with ties resolved in favor of the preceding run. This smoothing step is applied before kinematic synthesis.

### 2.7. Sensor-Aware Generative Framework Algorithm

Algorithm 1 summarizes the proposed sensor-aware generative framework used throughout this study. The model is calibrated once from the real sessions and then reused across all reported experiments. Unless otherwise stated, each synthetic session is generated with length T=3000 frames, corresponding to 300 s of simulated behavior at Δt=0.1 s.
**Algorithm 1:** Proposed sensor-aware generative framework with explicit observation channel  1:**Input:** Paired reference and observed data $\left\{\left(x_{t},{\tilde{x}}_{t},z_{t}\right)\right\}$, sampling interval Δt, target length *T*, sensing regime c∈{LoS,NLoS}.  2:Segment the frame-level labels into runs $\left\{\left(s_{k},d_{k}\right)\right\}$ using Equation (8).  3:Estimate the run-level transition matrix $\hat{P}$ using Equation (11), and estimate the dwell-time distributions $\left\{D_{i}\right\}$ using Equation (13); enforce ${\hat{P}}_{ii}=0$ and renormalize each row over j≠i.  4:Compute the empirical frame-level occupancy $\pi^{\mathrm{real}}$ and calibrate the transition matrix P(α) using Equation (20).  5:Estimate the state-conditioned kinematic priors $\left\{L_{i},T_{i}\right\}$ and compute latent increment caps using Equations (33) and (34).  6:Estimate the distortion parameters $\left\{\Psi_{i,c}\right\}$ from the residual process defined in Equation (23) under the model in Equations (3) and (4), and compute distortion caps using Equation (36).  7:Generate a run-based state sequence $z_{1:T}$ using P(α) and $\left\{D_{i}\right\}$, then apply smoothing according to ${\tilde{z}}_{1:T}=S\left(z_{1:T}\right)$ via Equation (40).  8:Initialize $x_{1}$ and $\phi_{1}$.  9:Sample distortion $e_{1}∼p\left(e|{\tilde{z}}_{1},c\right)$ under Equations (3) and (4), and compute the capped distortion $e_{1}^{\mathrm{cap}}$ using Equation (37).10:Form the sensor-observed position ${\tilde{x}}_{1}\leftarrow x_{1}+e_{1}^{\mathrm{cap}}$.11:**for** t=2 to *T* **do**12:   Sample latent increments $\ell_{t}∼\hat{p}\left(L_{{\tilde{z}}_{t}}\right)$ and $\theta_{t}∼\hat{p}\left(T_{{\tilde{z}}_{t}}\right)$, and apply the caps in Equation (34).13:   Update the latent heading $\phi_{t}$ and latent position $x_{t}$ using Equations (38) and (39).14:   Sample distortion $e_{t}∼p\left(e|{\tilde{z}}_{t},c\right)$ under Equations (3) and (4), and compute the capped distortion $e_{t}^{\mathrm{cap}}$ using Equation (37).15:   Form the sensor-observed position ${\tilde{x}}_{t}\leftarrow x_{t}+e_{t}^{\mathrm{cap}}$.16:**end for**17:**Output:** Synthetic observed trajectory and labels ${\left\{\left({\tilde{x}}_{t},{\tilde{z}}_{t}\right)\right\}}_{t=1}^{T}$, together with the latent trajectory ${\left\{x_{t}\right\}}_{t=1}^{T}$.

### 2.8. Statistical Turing Test

We evaluate the realism of the proposed sensor-aware generative framework by comparing real and synthetic trajectories along two complementary dimensions: (i) agreement in behavioral-state occupancy and (ii) agreement in state-conditioned kinematic feature distributions. Together, these metrics assess whether the synthetic data reproduce both the prevalence of behavioral states and the local motion statistics observed in the real trajectories.

**State occupancy:** Let ${\tilde{z}}_{t}\in Z$ denote the final synthetic behavioral label at time index *t* after the smoothing step in Equation (40), and let *T* denote the total number of frames in a generated session. The synthetic frame-level occupancy of state i∈Z is defined as
  (42) $$\pi_{i}^{\mathrm{synth}}≜\frac{1}{T}\sum_{t=1}^{T}I\left\{{\tilde{z}}_{t}=i\right\}$$
  where I{·} denotes the indicator function. We quantify occupancy agreement by reporting the occupancy error vector $\pi^{\mathrm{synth}}-\pi^{\mathrm{real}}$, where $\pi^{\mathrm{real}}$ is the empirical frame-level occupancy computed analogously from the real data.

**State-conditioned KL divergence:** To assess agreement in local kinematic structure, we compare the state-conditioned distributions of step length and turning angle. Let f∈{ℓ,θ} denote the feature of interest. For each behavioral state i∈Z, we construct histograms for the real and synthetic samples of *f* using shared bin edges, yielding bin counts $n_{i,b}^{f}$ for the real data and ${\tilde{n}}_{i,b}^{f}$ for the synthetic data, where b=1,…,B. We set B=50 and determine the bin edges from the regime-pooled range of *f* so that divergence values are not confounded by changes in binning across LoS and NLoS conditions. We additionally verified that the qualitative conclusions remain stable over a moderate range of bin counts (e.g., B∈[40,60]).

To avoid zero-probability bins, we apply additive smoothing and normalize the histogram counts to obtain probability mass functions:

(43) $$p_{i,b}^{f}≜\frac{n_{i,b}^{f}+\epsilon }{\sum_{b^{\prime }=1}^{B}\left(n_{i,b^{\prime }}^{f}+\epsilon \right)},\;q_{i,b}^{f}≜\frac{{\tilde{n}}_{i,b}^{f}+\epsilon }{\sum_{b^{\prime }=1}^{B}\left({\tilde{n}}_{i,b^{\prime }}^{f}+\epsilon \right)},$$

where ϵ>0 is a small regularization constant (e.g., $10^{-12}$) [21,22]. Using the real distribution as the reference, we compute the Kullback–Leibler (KL) divergence

(44) $$D_{\mathrm{KL}}\;\left(p_{i}^{f}\;∥\;q_{i}^{f}\right)≜\sum_{b=1}^{B}p_{i,b}^{f}\log\;\left(\frac{p_{i,b}^{f}}{q_{i,b}^{f}}\right)$$

Smaller values of $D_{\mathrm{KL}}$ indicate closer agreement between the real and synthetic feature distributions within the corresponding behavioral state.

**Occupancy-weighted summaries:** Because some behavioral states occur much less frequently than others, unweighted aggregation can overemphasize discrepancies in rare states relative to their contribution to overall behavior. To obtain a summary measure that respects empirical state prevalence, we report an occupancy-weighted KL divergence:
  (45) $$D_{\mathrm{WKL}}^{f}≜\sum_{i\in Z}\pi_{i}^{\mathrm{real}}\;D_{\mathrm{KL}}\;\left(p_{i}^{f}\;∥\;q_{i}^{f}\right)$$
  where $\pi_{i}^{\mathrm{real}}$ denotes the empirical frame-level occupancy of state *i* in the data. Weighting by $\pi_{i}^{\mathrm{real}}$ ensures that the overall summary reflects the importance of each state while preventing rare-state divergences from disproportionately dominating the realism assessment.

### 2.9. Temporal Realism via Mean-Squared Displacement

Agreement in marginal kinematic statistics does not, by itself, ensure that a synthetic trajectory reproduces the temporal organization of real behavior. To evaluate displacement dynamics across multiple time scales, we therefore compute the MSD as a function of lag. For a discrete trajectory ${\left\{x_{t}\right\}}_{t=1}^{T}$ sampled at temporal interval Δt in Seconds, the MSD at lag *k* frames, corresponding to physical lag τ=kΔt, is defined as

(46) $$\mathrm{MSD}\left(k\right)≜\frac{1}{T-k}\sum_{t=1}^{T-k}{∥x_{t+k}-x_{t}∥}_{2}^{2}$$

where the resulting quantity is expressed in Square Meters. The same definition is applied to observed trajectories by replacing $x_{t}$ with ${\tilde{x}}_{t}$.

For each sensing regime, MSD curves are computed for all real sessions and synthetic trajectories and then averaged within each group. Variability at each lag is summarized by the standard deviation across trajectories. Let ${\bar{\mathrm{MSD}}}_{\mathrm{real}}\left(k\right)$ and ${\bar{\mathrm{MSD}}}_{\mathrm{syn}}\left(k\right)$ denote the corresponding mean MSD curves for the real and synthetic sets, respectively. To summarize the degree of temporal agreement between the two, we report the root-mean-square error (RMSE) between the mean curves over lags k=1,…,K:

(47) $${\mathrm{RMSE}}_{\mathrm{MSD}}≜\sqrt{\frac{1}{K}\sum_{k=1}^{K}{\left({\bar{\mathrm{MSD}}}_{\mathrm{syn}}\left(k\right)-{\bar{\mathrm{MSD}}}_{\mathrm{real}}\left(k\right)\right)}^{2}}$$

Smaller values of ${\mathrm{RMSE}}_{\mathrm{MSD}}$ indicate closer agreement in multi-scale displacement dynamics between the synthetic and real trajectories.

### 2.10. Synthetic Cohorts and Baselines

Once calibrated, the proposed framework can generate synthetic sessions for controlled proof-of-concept analyses. In the present study, this capability is used for trajectory-level realism assessment, controlled downstream robustness testing, and baseline comparisons that isolate the effect of sensor-aware conditioning; it should not be interpreted as a fully validated replacement for experimentally acquired cohorts.

**Trajectory-level sessions for qualitative analysis and Turing-test evaluation:** We generate sensor-aware synthetic sessions of length T=3000 frames under each sensing regime (LoS and NLoS) and compare them with real sessions using the realism metrics in Section 2.8. The choice T=3000 matches the evaluation horizon and provides sufficient state coverage for histogram-based comparisons.
**Controlled synthetic cohorts for downstream robustness testing:** To support controlled downstream analysis, we generate a pool of synthetic sessions using the calibrated framework parameters. Each synthetic session is generated from a distinct random seed and is treated as one independent sample for the robustness benchmark. This design supports pilot testing, sensitivity analysis, and methodological stress-testing while remaining aligned with the Reduction principle of the 3Rs framework [3]. To construct a downstream binary classification benchmark, each synthetic session is assigned a label y∈{0,1}, where y=0 denotes an unperturbed synthetic session and y=1 denotes a perturbed synthetic session generated by applying a mild disease-inspired perturbation to the motion model. Specifically, the perturbed class is created by combining a random speed reduction of 2–12%, relabeling 0.3–1.2% of exploring frames as feeding, and adding positional jitter with standard deviation 0.001–0.006 m. Because disease labels are not available for the real UWB sessions, this benchmark is used only to evaluate domain-shift robustness in a controlled synthetic setting. It should therefore be interpreted as a methodological test of sensitivity to sensing-induced distortions rather than as evidence of biological or clinically validated phenotypes. From this labeled pool, we construct a balanced synthetic cohort of size $N_{\mathrm{syn}}=600$, with 300 sessions per class. Training and testing use disjoint subsets generated from non-overlapping random seeds, ensuring that no synthetic trajectory appears in more than one split. We then compare two classifier settings: a condition-agnostic baseline and a sensor-aware classifier that includes sensing regime as an explicit feature.
**Sensor-aware versus sensor-agnostic baselines:** To isolate the contribution of explicit sensing-regime modeling, we compare the proposed sensor-aware framework, which uses the regime-conditioned observation channel in Equations (3) and (4), with a condition-agnostic baseline that omits sensing regime as an explicit covariate in downstream classification. Under this baseline, classifiers are trained on synthetic sessions generated under one sensing regime and evaluated on sessions generated under the other, thereby testing cross-domain transfer under LoS/NLoS shift. This comparison assesses whether explicit sensor awareness improves robustness when the observation process differs across training and test conditions [11,12,13].

## 3. Results

### 3.1. Statistical Realism Evaluation of the Generative Framework

**Figure 2:**
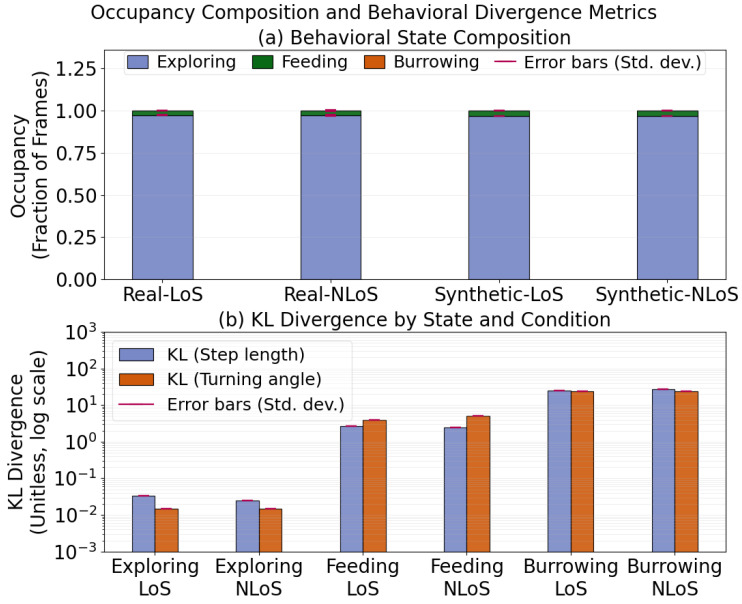
Per-state statistical Turing metrics. (**a**) Behavioral-state occupancy (fraction of frames) for real and synthetic sessions under LoS and NLoS conditions. (**b**) State-conditioned KL divergence between real and synthetic distributions for step length and turning angle (log scale), reported by behavioral state and sensing regime. Bars indicate mean values; error bars denote standard deviation where available.

**Figure 3:**
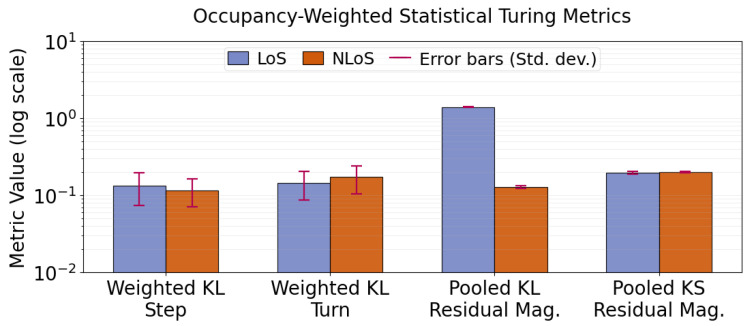
Occupancy-weighted statistical Turing metrics. Occupancy-weighted KL divergence for step length and turning angle aggregates per-state divergences using real-state occupancy as weights. Residual-magnitude agreement is evaluated by pooled KL divergence and the KS statistic applied to $∥e_{t}{∥}_{2}$ within each sensing regime. Error bars denote standard deviation across random seeds.

Using the framework in Section 2.8, we assess the realism of the proposed sensor-aware generative framework by comparing real sessions with synthetic sessions generated under matched sensing regimes (LoS and NLoS). Because this evaluation is based on four UWB sessions, consisting of two LoS and two NLoS recordings, the results should be interpreted as proof-of-concept evidence rather than as a generalizable validation across animals, environments, or cage configurations. Figure 2 reports the per-state Turing metrics, namely frame-level occupancy and state-conditioned KL divergence for step length and turning angle. Figure 3 summarizes occupancy-weighted KL divergences together with pooled residual-magnitude divergences, measured using both KL divergence and the Kolmogorov–Smirnov (KS) statistic, under each sensing regime. Unless noted otherwise, all synthetic summaries are computed from an ensemble of independently seeded synthetic sessions for each sensing regime, and reported values are shown in the figures as mean ± standard deviation across seeds.

The numerical values corresponding to the occupancy, state-conditioned KL, occupancy-weighted KL, pooled residual KL, and pooled KS metrics are incorporated directly into Figure 2 and Figure 3 through plotted bar values and associated error bars. In such a low-support setting, histogram-based KL estimates become highly sensitive to bin-level sparsity and tail fluctuations, so even modest absolute mismatches can produce numerically large divergence values. For this reason, the occupancy-weighted summaries in Figure 3 provide the more representative aggregate assessment of overall realism, because they account for the empirical contribution of each state to the full trajectory distribution. For residual magnitudes, we report both KL divergence and the KS statistic because they capture complementary aspects of agreement: KL divergence is sensitive to concentration and tail mismatch, whereas the KS statistic measures global distributional separation in a scale-robust manner. Taken together, these results indicate that the largest rare-state discrepancies are localized to a very low-occupancy regime and do not overturn the broader agreement observed in occupancy, weighted kinematic summaries, and MSD-based temporal realism.

#### MSD Evaluation of Temporal Realism

**Figure 4:**
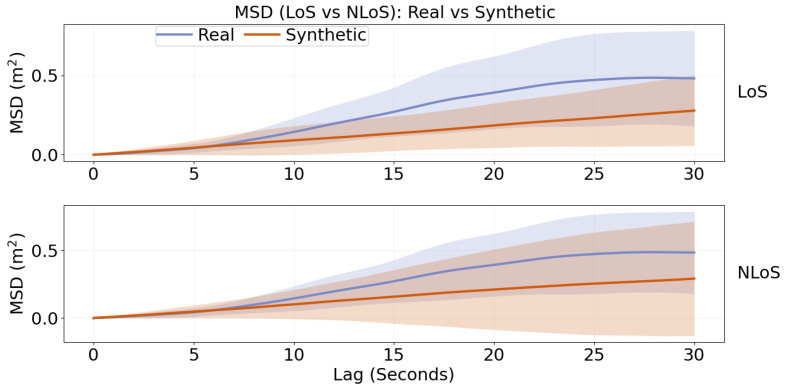
Mean-squared displacement (MSD) of real and synthetic trajectories under LoS and NLoS conditions. Curves are averaged across sessions and seeds; shading indicates ±1 standard deviation.

To assess temporal realism beyond marginal feature agreement, we use the MSD metric defined in Section 2.9. Figure 4 compares real and synthetic MSD curves under LoS and NLoS over lags from Δt to 30.0 s. The corresponding RMSE values are reported directly here as $5.52\times 10^{-2}$ Square Meters for LoS and $6.06\times 10^{-2}$ Square Meters for NLoS, providing numerical support for Figure 4 comparison and indicating good agreement in multi-scale displacement dynamics.

### 3.2. Cross-Domain Classifier Degradation Under Domain Shift

We next examine sensing-induced domain shift in downstream inference using a controlled synthetic experiment. Specifically, a classifier is trained on synthetic cohorts generated under one sensing regime and evaluated on cohorts generated under the other, thereby providing a controlled test of domain-shift effects rather than evidence of biological or translational utility. Table 1 contrasts the condition-agnostic baselines with the sensor-aware model on the synthetic unperturbed-versus-perturbed benchmark under LoS/NLoS domain shift. Under the condition-agnostic baseline, cross-domain performance degrades substantially, particularly at the fixed operating point used for thresholded prediction: training on LoS and testing on NLoS yields AUC=0.974 with balanced accuracy 0.594, whereas training on NLoS and testing on LoS yields AUC=0.901 with balanced accuracy 0.500 (Table 1). These results indicate that sensing-regime-specific distortions can shift the induced feature distribution enough to weaken naive cross-regime transfer, even when the underlying class-defining perturbation is held fixed.

### 3.3. Sensor-Aware Classifier Mitigates Cross-Domain Failure

We next evaluate whether explicit conditioning on sensing regime improves downstream robustness. When sensing condition is included as a covariate, performance remains high on the mixed-domain test set (LoS + NLoS; AUC=0.995), as well as on the LoS-only (AUC=0.998) and NLoS-only (AUC=0.993) subsets (Table 1). Relative to the condition-agnostic baselines, these results indicate that sensor awareness substantially mitigates domain-induced performance degradation in these datasets.

**Classifier protocol:** Synthetic cohorts are generated using non-overlapping random seeds across splits so that no trajectory appears in more than one set. Each session is represented using summary trajectory features derived from state occupancy, step-length statistics, turning-angle statistics, residual-magnitude statistics, and MSD-based temporal descriptors. The classifier is implemented as a regularized logistic-regression model using the same preprocessing, feature set, and decision-thresholding procedure across all evaluation settings. The sensing regime is included as an additional covariate in the sensor-aware setting and omitted in the condition-agnostic baselines. For cross-domain transfer, the classifier is trained on synthetic sessions generated under one sensing regime and evaluated on sessions generated under the other. For mixed-domain evaluation, the test set includes samples from both regimes. AUC is computed from decision scores, whereas accuracy and balanced accuracy are computed using a fixed decision threshold. Because the benchmark is generated synthetically from controlled perturbations, these results should be interpreted as a domain-shift robustness test rather than as biological phenotype classification.

### 3.4. Representative Trajectories: Qualitative Realism

**Figure 5:**
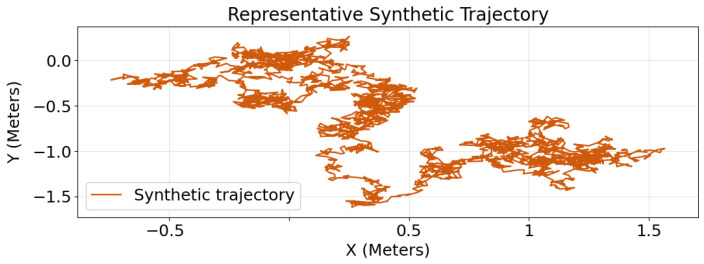
Representative synthetic trajectory generated by the sensor-aware generative framework. The trajectory shows plausible spatial coverage and state-dependent motion patterns consistent with the real sessions. Axes are expressed in Meters.

In addition to the quantitative realism metrics, we examine representative trajectories to assess qualitative agreement between real and synthetic motion patterns. Figure 5 shows a representative trajectory generated by the sensor-aware generative framework. The figure provides qualitative evidence that the proposed model reproduces several visually important properties of the real sessions, including plausible spatial coverage, extended exploring segments, and localized feeding and burrowing events. It also shows that the synthetic trajectory preserves coherent state organization over time rather than degenerating into implausible fragmentation or uniformly random motion. Thus, Figure 5 complements the quantitative realism metrics by illustrating that the proposed framework captures not only distributional agreement but also an interpretable trajectory structure that is visually consistent with the real UWB recordings.

**Figure 6:**
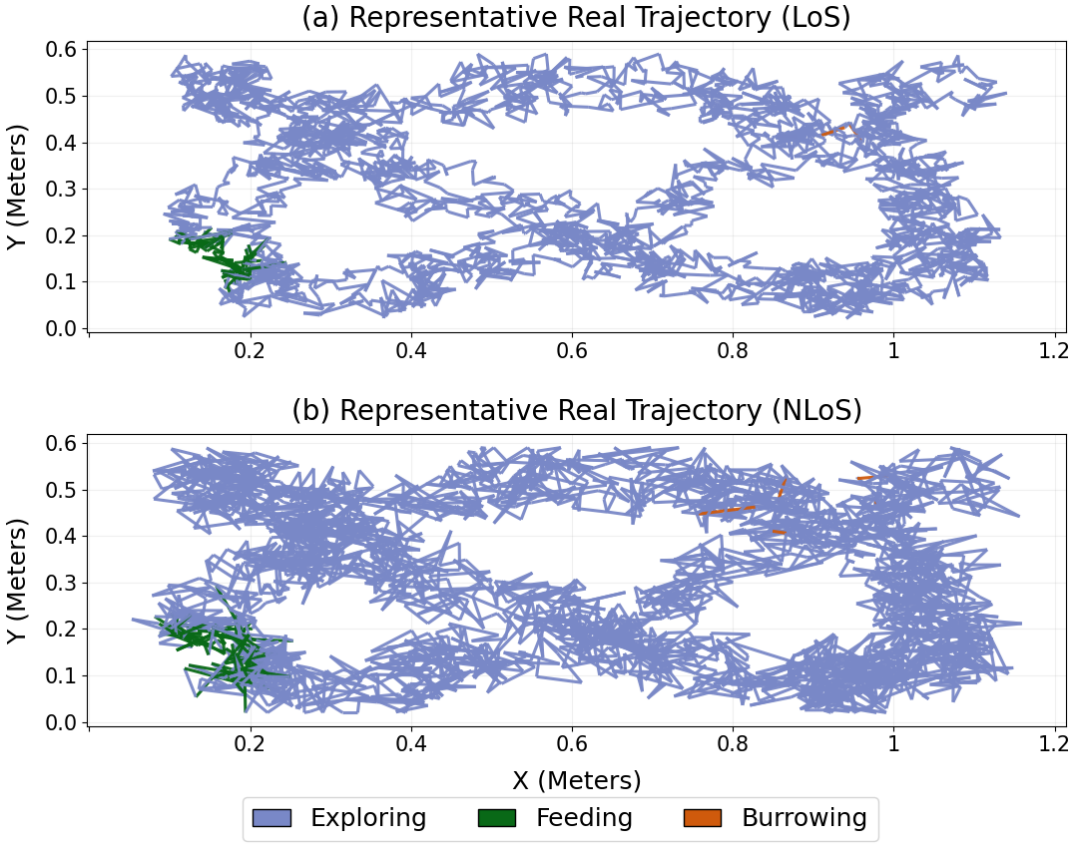
Representative real trajectories under (**a**) LoS and (**b**) NLoS sensing conditions, colored by behavioral state (exploring, feeding, and burrowing). Axes are expressed in Meters.

Figure 6 shows example real trajectories under LoS and NLoS, with positions colored by behavioral state. Under LoS conditions (Figure 6a), trajectories show smooth spatial continuity and clear state transitions, with extended exploring segments interspersed with localized feeding or burrowing events. By contrast, the NLoS trajectory (Figure 6b) exhibits greater positional irregularity and occasional abrupt deviations consistent with sensing-induced distortion while preserving the overall behavioral structure. All axes are expressed in Meters.

## 4. Discussion

### 4.1. Methodological Perspective

The central methodological contribution of this work is a sensor-aware factorization for in silico ethology that explicitly separates latent behavioral generation from proxy-referenced observation modeling. In the proposed formulation, an ethology-driven latent trajectory $\left\{x_{t}\right\}$ is mapped into the observed UWB domain $\left\{{\tilde{x}}_{t}\right\}$ through a sensing-regime-dependent observation channel conditioned on LoS and NLoS propagation (Section 2.5.4). This separation is important because it avoids conflating behavioral dynamics with sensing artifacts, thereby allowing the generative model to preserve interpretable latent motion structure while reproducing regime-specific observation distortions. Because a ground-truth trajectory is unavailable in UWB-only experiments, the reference path $x_{t}$ must be interpreted as a smoothed UWB-derived proxy rather than a directly observed physical truth (Section 2.5.1). Accordingly, the residual process used to parameterize the observation channel should be understood as a proxy-referenced distortion model rather than as a direct estimate of localization error with respect to independent ground truth. This distinction limits claims about absolute localization-error validity and motivates future validation against synchronized external references such as video tracking.

### 4.2. Interpretation of Realism Metrics

The statistical Turing results indicate that the proposed framework captures the dominant structure of the real data at multiple levels. At the behavioral level, the semi-Markov layer reproduces frame-level occupancy through the interaction of run-level visitation frequencies and state-specific dwell-time statistics, as formalized by Equations (15) and (16) and enforced through the calibration step in Equation (20). At the kinematic level, the state-conditioned priors reproduce the principal mass of the step-length and turning-angle distributions. Because occupancy and state-conditioned kinematic distributions are related to quantities used during model calibration, these metrics are interpreted primarily as internal realism checks; complementary MSD analysis and downstream domain-shift testing provide additional evaluation beyond the direct calibration targets. The largest discrepancies arise in sparse states, particularly where histogram-based divergence estimates become variance-limited due to low sample support (Section 2.8). This behavior is consistent with the occupancy-weighted summaries, which show that large per-state divergences in rare states do not necessarily imply poor overall realism when those states occupy only a small fraction of frames.

### 4.3. Temporal Realism Beyond Marginal Agreement

Agreement in marginal kinematic distributions does not guarantee realistic temporal structure, which is why MSD provides an important complementary test. By evaluating displacement across multiple lags, the MSD analysis probes whether the synthetic trajectories reproduce the temporal organization of movement rather than merely its one-step statistics. The agreement between real and synthetic MSD curves in Figure 4, together with the low RMSE values reported for both LoS and NLoS, suggests that the proposed model captures multi-scale displacement dynamics with useful fidelity in the present dataset. This result is notable because the generator is intentionally simple and interpretable, relying on semi-Markov state dynamics and empirical state-conditioned kinematic priors rather than on a high-capacity sequence model.

### 4.4. Why Sensor-Awareness Matters

A central finding of this study is that the sensing regime is not a minor nuisance factor but a structured source of distribution shift. Through the observation channel defined in Equations (3) and (4), LoS and NLoS conditions induce distinct distortions in the observed trajectory space, which in turn alter the distributions of downstream motion features. Under this view, cross-regime generalization should not be interpreted as robustness to small additive noise, but rather as robustness to a systematic shift in the observation process. This distinction is important because it reframes sensing as part of the generative mechanism itself. The results suggest that models trained without explicit awareness of sensing conditions are vulnerable not simply to measurement variability but to domain mismatch at the level of the observation distribution.

### 4.5. Downstream Inference Under Domain Shift

The classifier experiments support this interpretation within a controlled synthetic benchmark. When sensing conditions are ignored, performance degrades under LoS↔NLoS transfer, indicating that domain-specific distortions can dominate the induced feature space and undermine naive cross-regime generalization. By contrast, including sensing condition as an explicit covariate substantially mitigates this failure mode in the present dataset. Because the class labels are synthetically perturbed rather than biologically validated disease labels, these results should be interpreted as evidence of improved robustness to sensing-domain shift, not as evidence of biological or translational classification utility. More broadly, the experiment supports the modeling principle that synthetic data generation for behavioral analysis should preserve not only latent dynamics but also the structure of the sensing process through which those dynamics are observed.

### 4.6. Limitations

Several limitations should be acknowledged. First, the state-conditioned priors in Equation (29) are empirical and estimated from only four sessions, consisting of two LoS and two NLoS recordings. This limited empirical basis restricts the breadth of ethological variation represented by the model and constrains generalization to broader behavioral repertoires, additional animals, cage configurations, and environments. Although variability across independently seeded synthetic runs is reported in the main figures, the limited number of real sessions prevents a comprehensive leave-one-session-out or cross-animal sensitivity analysis. Such analyses should be included in future studies with larger datasets to quantify dependence on individual sessions. Second, the kinematic generator assumes conditional independence of $\left(\ell_{t},\theta_{t}\right)$ across time given behavioral state and therefore does not explicitly capture longer-range temporal dependencies, higher-order motion motifs, or subject-specific behavioral style. Third, rare-state statistics remain data-limited and can inflate divergence-based metrics because low-support histogram estimates are sensitive to sparsity and tail fluctuations, even when the corresponding state contributes little to aggregate realism. Fourth, although the observation channel captures regime-dependent bias and heavy-tailed residual variation, UWB observation distortions may also exhibit temporal autocorrelation, spatial dependence, and burst-like NLoS artifacts that are not explicitly modeled here. Fifth, the present framework is sensor-aware and trajectory-level, but it is not intended to represent a full biological digital twin of the animal; rather, it models behavioral dynamics and regime-dependent observation distortion within the scope of the recorded locomotor data. Finally, the current generator operates in planar space and does not explicitly encode arena boundaries, obstacle interactions, or task-specific environmental structure unless such constraints are imposed after synthesis.

### 4.7. Outlook

Despite these limitations, the proposed framework provides a principled and interpretable probabilistic baseline for sensor-aware in silico ethology. A natural next step is to replace the empirical sampling procedure in Equation (29) with a learned conditional sequence model capable of capturing longer-range temporal dependencies while retaining the explicit observation-layer factorization developed here. More broadly, sensor awareness could be preserved either by conditioning directly on the sensing regime c∈{LoS,NLoS} or by learning a latent domain variable that encodes signal integrity and measurement quality [16]. Such extensions would preserve the central insight of this work: realistic behavioral simulation requires modeling not only how animals move, but also how sensing systems transform that motion into the data domain used by downstream inference.

## 5. Conclusions

We have presented a sensor-aware generative framework for in silico ethology that explicitly separates latent behavioral synthesis from a sensing-regime-dependent observation channel. This factorization enables controlled generation of observed-domain synthetic trajectories that preserve ethological structure while reproducing proxy-referenced observation distortions associated with LoS and NLoS conditions. Across the proposed realism metrics and MSD-based temporal evaluation, the generated trajectories exhibit close agreement with real data in behavioral occupancy, kinematic distributions, and multi-scale displacement dynamics.

A central finding of this study is that the sensing regime induces structured shifts in the observed trajectory domain that can substantially degrade cross-domain classifier performance when the sensing condition is ignored. By contrast, incorporating a sensing regime as an explicit covariate mitigates this failure mode in the present dataset, indicating that sensor awareness is not merely a modeling refinement but a practically important design principle for mixed-regime behavioral inference.

More broadly, the proposed framework provides an interpretable probabilistic baseline for sensor-aware in silico ethology under controlled conditions, while the ability to generate synthetic cohorts may support pilot experimentation and robustness analysis; such applications should be interpreted as preliminary in the present proof-of-concept setting and require validation on larger and more diverse datasets. This remains aligned with the reduction principle of the 3Rs. Future work will extend the framework using larger multi-animal datasets and learned sequence models capable of capturing longer-range temporal dependencies while preserving an explicit sensor-aware observation layer.

## Figures and Tables

**Table 1:** Classifier performance on the synthetic unperturbed-versus-perturbed benchmark under LoS/NLoS sensing-domain shift. The sensor-aware classifier includes sensing regime as an explicit covariate, whereas the condition-agnostic baselines omit this information and are evaluated under cross-domain transfer. AUC is computed from decision scores, while accuracy and balanced accuracy are computed using a fixed decision threshold.

Evaluation Setting	AUC	Accuracy	Balanced Accuracy
Sensor-aware: mixed-domain test (LoS + NLoS)	0.995	0.973	0.973
Sensor-aware: test on LoS only	0.998	0.973	0.973
Sensor-aware: test on NLoS only	0.993	0.973	0.973
Condition-agnostic: train LoS, test NLoS	0.974	0.594	0.594
Condition-agnostic: train NLoS, test LoS	0.901	0.500	0.500

## Data Availability

Processed, de-identified datasets and all analysis code that support this study are publicly available at the Open Science Framework (OSF): https://osf.io/psme9 (accessed on 7 May 2026). The repository includes final figures, a provenance manifest, processed data (errors, state sequences, transition matrices, summary metrics), and minimal analysis scripts. Raw data are not included.
